# Labels Direct Infants’ Attention to Commonalities during Novel Category Learning

**DOI:** 10.1371/journal.pone.0099670

**Published:** 2014-07-11

**Authors:** Nadja Althaus, Denis Mareschal

**Affiliations:** 1 Centre for Brain and Cognitive Development, Department of Psychological Sciences, Birkbeck, University of London, London, United Kingdom; 2 Department of Experimental Psychology, University of Oxford, Oxford, United Kingdom; Goldsmiths, University of London, United Kingdom

## Abstract

Recent studies have provided evidence that labeling can influence the outcome of infants’ visual categorization. However, what exactly happens during learning remains unclear. Using eye-tracking, we examined infants’ attention to object parts during learning. Our analysis of looking behaviors during learning provide insights going beyond merely observing the learning outcome. Both labeling and non-labeling phrases facilitated category formation in 12-month-olds but not 8-month-olds (Experiment 1). Non-linguistic sounds did not produce this effect (Experiment 2). Detailed analyses of infants’ looking patterns during learning revealed that only infants who heard labels exhibited a rapid focus on the object part successive exemplars had in common. Although other linguistic stimuli may also be beneficial for learning, it is therefore concluded that labels have a unique impact on categorization.

## Introduction

In recent years, there has been an intriguing debate focusing on the question of the possible interactions between labeling and categorization in infancy. Indeed, by the end of their first year, infants have both sophisticated language processing skills (e.g., [1], [2], [3], [4]) and categorization abilities [5], [6], [7], [8], [9], [10], [11]. This raises the question of what role language plays in shaping category formation. The idea that language may affect the way humans categorize objects has a long history. For example, James [12] suggested that associating different wines with their names – more discriminable than the tastes themselves – may help to distinguish them. In the 20^th^ century, the idea of linguistic determinism [13] arose as the most extreme form of an impact of language on cognition. While this extreme position has, on balance, not been supported by empirical evidence, the possibility of interactions between language and object-processing, specifically in children and infants, has recently received support [14], [15], [16], [17].

Waxman and Markow [15] conducted a seminal study in which 12- to 13-month-old infants were familiarized with sets of toys, and either provided with labeling phrases (e.g., “Look, an avi!”) or non-labeling phrases (e.g., “Look what’s here!”). On a test trial that presented a novel within-category stimulus alongside an out-of-category object, preference for the out-of-category stimulus was taken as an indicator of successful categorization. The rationale underlying this familiarization/novelty preference procedure is that in order to exhibit a preference for the novel item, the subject must both recognize that the within-category stimulus is similar to the familiarized exemplars, and at the same time *reject* the out-of-category stimulus as being similar to those items. Labeling did not cause the infants to increase their preference for an out-of-category object on test in the case of “basic-level” categories (e.g., cows vs. dinosaurs; cf. [18]), where infants were already successful at category formation in the “No Label” condition. When “superordinate-level” categories (e.g., animals vs. vehicles) were used, however, only infants in the “Label” condition reliably preferred the out-of-category object. The authors’ interpretation of this was that labels are “invitations to form categories”, and they hypothesized that labels may “highlight commonalities”. Using screen-based presentation of animal pictures, those findings were extended to age groups as young as 3 to 4 months [16], [19]. Although for very young infants facilitation was also achieved using primate vocalizations instead of labels [20], the effect appears to be specific to speech-like stimuli by 6 months [21], [22], [16]. In contrast, unsystematic labeling with different words did not cause any facilitation effects [23].

Plunkett, Hu, and Cohen [24] presented further work highlighting the constructive effects of labels on categorization. In their study, 10-month-olds provided with identical labels for each familiarization exemplar formed a single category over the same set of stimuli that infants divided into two groups when familiarized in silence. This merging of visual subcategories did not occur when two distinct labels were paired systematically with the two subsets, indicating that infants relied on the label’s identity to form categories. In contrast to these generally positive effects, Robinson and Sloutsky [25] reported disruptive effects of labels. In this work, 12-month-olds familiarized with a sequence of cat images increased novelty preference for a bear image only when familiarization took place in silence, not when the pictures were accompanied by names or novel sounds. The authors attributed this outcome to auditory overshadowing. The result is consistent with previously reported results from audio-visual integration studies with children, and studies on processing speed with infants [26]. Furthermore, unfamiliar auditory input causes more overshadowing than familiar auditory signals [26]. The authors argued, however, that initial auditory overshadowing could eventually contribute to category learning by reducing the perception of dissimilarities in the visual exemplars [27].

While intriguing, the studies discussed above do not – together – provide a consistent picture of the impact of labeling on categorization. For example, it remains unclear whether differences between the studies reported by Robinson and Sloutsky [25] and by Waxman and colleagues arise from methodological differences.

More importantly however, these studies do not address the question of *how* labeling impacts on category formation. Category formation is an incremental process heavily dependent on the nature of the familiarization stimuli [7], [9], [11], [28] and even their order [29]. This implies that what happens during learning is of crucial interest. In particular, Waxman and Markow’s [15] hypothesis that labels direct attention to commonalities has not been tested directly. This is what we aim to do in the present work.

A labeling event (i.e., perceiving an object-word pair) triggers many cognitive processes, and they may interact with visual object processing in different ways. One possibility is that simultaneous bottom-up processes (visual and auditory) interfere with each other (e.g., because of a lack of processing resources) and as a result processing (in one or both modalities) is attenuated [25] (Hypothesis a.). Another possibility is that labeling merely makes encountering the object more *salient,* leading the infant to process stimuli in more detail and possibly pick up patterns more quickly – via attentional mechanisms that are unrelated to the identity of the additional stimulus [23] (Hypothesis b.). In this case, any beneficial effects would be similar to those caused by other speech, or even non-speech auditory stimuli, although labels may prove to be particularly effective. Yet another scenario is that the interactions in a labeling event occur at a higher level (Hypothesis c.). In a category learning context, having an object-label association may help infants to re-activate visual representations of previously seen exemplars, or even already established prototypes, and could thereby allow faster category encoding. In this case, beneficial effects seen with labels should be distinct from the effects other (speech or auditory) stimuli have on category learning.

For adults, Lupyan and colleagues found that labeling facilitated category formation even when the labels were redundant [30]. Lupyan [31] further reported decreased memory for individual items in the context of labels. Stimulus encoding appeared to have shifted towards the prototype of the labeled category. Lupyan and Spivey [32] found that subjects were better at detecting a target probe occurring in spatial proximity to one of several stimuli if their attention had been directed to these stimuli through naming. Both Lupyan’s [31] and Lupyan and Spivey’s [32] findings suggest that labels have a top-down effect on earlier visual processes. Effects like these may also occur when infants process objects and words. For example, Gliga, Volein, and Csibra [33] showed in a study measuring induced EEG gamma-band activation that having previously heard an object being labeled modulated 12-month-olds’ visual processing of that object. The increased gamma-band activation was only found for familiar objects with known associated words and novel objects that had been named in a preceding play session – neither familiar objects whose names were unknown, nor novel, unnamed objects elicited this effect. In a phonological priming task using picture primes, Mani and Plunkett [34] demonstrated that even infants as young as 18 months implicitly generate phonological representations upon seeing a picture for which they know a word. Both Mani and Plunkett’s [34] implicit naming study and the increased EEG gamma-band activation found by Gliga et al. [33] for previously labeled objects suggest that having stored a label for an object changes processing of the object even when the label is absent.

What really happens when infants learn a category in the presence of labels therefore needs to be investigated at a much more fine-grained scale than previous studies allow. One way of gaining insight into the interaction between labeling and categorization is to consider how hearing labels changes infants’ online processing of the visual objects *while they are engaged in learning* about a novel category. It is not enough merely to study the behavioral outcome of learning (e.g., preferential looking to a test item after familiarization). In addition it is necessary to observe *what features* infants attend to while they are learning. Thus, we present a study that aims to shed light on the question of how labels impact on the process of *category formation* (as opposed to outcomes) by using eye tracking during learning of a novel object category. This category involves spatially separate features which represent “commonalities” between exemplars as well as variable object parts. Tracking the amount of attention infants directed at individual object parts across the learning phase allowed us to test explicitly Waxman and Markow’s [15] hypothesis that labels “highlight commonalities”. Presenting the infants with a novel, unfamiliar category allowed us to pick up differences in processing between learning in the presence vs. absence of labels from the start of category formation; i.e., at a point where uncertainty about feature variability (and by extension, about diagnosticity for category membership) is highest.

By comparing four different conditions – (i) category formation in silence (Visual-only condition), (ii) in the presence of labeling phrases (Label condition, e.g. “Look at the Timbo!”) or (iii) non-labeling phrases (No Label condition, e.g. “Look at this!”) in Experiment 1, and (iv) unfamiliar, nonlinguistic sounds (Sound condition) in Experiment 2– we were further able to determine whether the observed effects were due to the addition of a signal in a different modality in general, the addition of speech specifically, or the addition of a novel label.

Since our target was to assess category formation during learning, we presented infants with a sequence of single objects from the target category in a familiarization procedure, during which *continuous category formation* processes could be observed. In particular, we were interested in infants’ responses to each object at the *start* of the trial. The categorization behavior we aimed to tap into during learning is a *fast* response occurring as soon as exposure to the visual object begins. In adults, categorization specific effects are found in ERPs from 80 ms onwards [35] and even when controlling for task-related priming effects ERP components after 200 ms are modulated by category assignment [36], indicating that the assignment of a newly perceived stimulus to a category has happened by this stage. Quinn, Westerlund and Nelson [37] as well as Grossmann, Gliga, Johnson and Mareschal [38] provided neural correlates of categorization in 6-month-olds which indicate that even in these young infants neural responses to familiar vs. novel categories diverge between 300 and 500 ms. Here, however, the category formation phase was also used to familiarize infants with the target category in order to elicit (in the case of successful learning) preferential looking after training. Every object presented during familiarization was therefore displayed for 5000 ms. Although we provide an initial analysis of behavior exhibited during the entire trial, we will see below that it is at the beginning of visual exposure that the impact of labels can be observed.

The familiarization phase was followed by a novelty preference test, a paired presentation of a consistent within-category object together with an out-of-category object, where we expected successful categorization to be reflected in a preference for the out-of-category object.

In Experiment 1 we familiarized 8- and 12-month-old infants with the target category in silence, with labeling phrases or with non-labeling phrases. Based on the existing literature (e.g., [16], [19]), we predicted that labeling would facilitate categorization in both 8- and 12-month-olds, and that infants in the Label condition would spend longer fixating the commonalities between objects than in the other conditions. We also predicted – in line with Waxman and Markow’s [15] finding – that categorization would not be facilitated in the No Label condition, implying that facilitation in the Label condition is dependent on the presence of novel labels.

## Experiment 1

### Method

#### Participants

Fifty 12-month-olds (mean age 359 days; 32 girls) and 54 8-month-olds (mean age: 252 days; 22 girls) participated in this study. Nine additional 12-month-olds and 10 additional 8-month-olds were tested, but not included in the analysis due to a failure to reach the looking time criterion of 5 or more familiarization trials with measured looking time data (14 infants), failure to calibrate successfully (4 infants) or technical problems (1 infant). Infants were randomly assigned to one of three conditions – Visual-only (12-month-olds: N = 17, 8-month-olds: N = 19), Label (12-month-olds: N = 17, 8-month-olds: N = 18) or No Label (12-month-olds: N = 16, 8-month-olds: N = 17). Only infants with English as their main language spoken at home were included. Infants were recruited via adverts in local parenting magazines and came mostly from the Greater London area.

#### Ethics statement

Informed consent was obtained in writing from parents or caregivers and all investigations were conducted according to the principles expressed in the Declaration of Helsinki, as well as the ethical principles of the British Psychological Society. Ethical approval was obtained by the Birkbeck School of Psychology Ethics Committee (Birkbeck Psychology Ethics Approval Certificate number 7806).

#### Stimuli & Design

**Figure 1:**
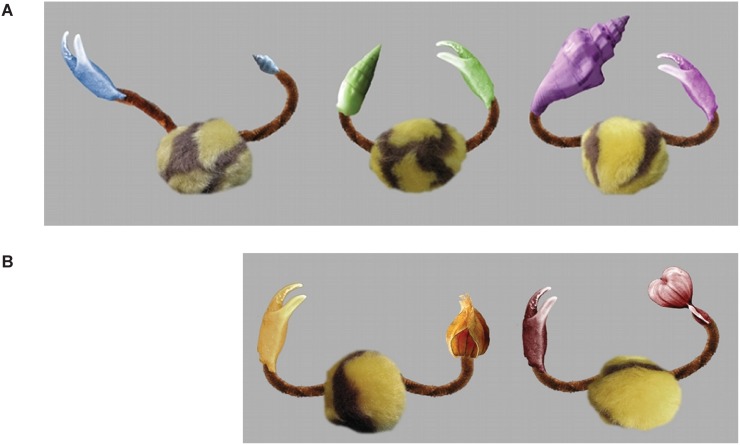
Stimuli. Panel A shows examples of familiarization stimuli, and panel B examples of out-of-category test stimuli.

Examples of visual stimuli are depicted in Figure 1. The visual target category objects (“Timbos”, Figure 1A) consisted of 5 parts: a ball, a claw and a shell (which were all possible targets in the looking time analyses) as well as two arm segments. All object parts were cut out from photographs of real objects and manipulated using the *GNU* image manipulation program (Gimp). The fluffy yellow and brown “ball” was highly similar between objects as it only varied in patterning, whereas the claw and shell differed in color between exemplars, and the shell also differed in shape and size. There were three different types of “shell”: large, small and medium (the latter being identical in pixel volume to the claw). The three shell types also differed in shape (see Figure 1A). Within one exemplar the claw and shell colors were made as visually similar as possible so as not to introduce differences in saliency. In addition, the arm position varied across objects. Together, the claws and shells made up the *high-variability* object parts. The ball was considered the *low-variability* part as it did not change in position, colour or shape – solely the patterning was varied in order to make sure infants would *discriminate* between exemplars, which is an important prerequisite for categorization. During the familiarization phase, half the Timbos were displayed on the left side, the other half on the right side of the screen. All Timbos were depicted against a medium-luminance grey background. The relative position of claw and shell on the left/right side of the object was counterbalanced, as was the position of claw and shell relative to the centre of the screen.

The category boundary test stimuli (see Figure 1B) were similar to the familiarization Timbos, but differed in one object part. Either the claw or the shell was replaced by a plant part (one of three flowers or a physalis fruit). The identity of the novel part was counterbalanced across subjects, to ensure that systematic preferences could not arise due to the attractiveness of one individual object part. On test, the target object was depicted alongside a novel Timbo (i.e., with ball, claw and shell). Across these two objects, claw, shell and plant part colors were the same (and different from all familiarization exemplars). The position of the target object on the left or right half of the screen was counterbalanced across subjects.

The auditory stimuli in the Label condition consisted of the phrases “Look at the Timbo!” (trials 1–3, 5–7) and “Do you see the Timbo?” (trials 4, 8). Phrases used in the No Label condition were “Look at this!” (trials 1–3, 5–7) and “Do you see this?” (trials 4, 8). All phrases were pre-recorded by a female native speaker of British English, using an infant-directed tone of voice.

#### Procedure

After a warm-up phase in the Babylab’s reception area, infants were seated in the caregiver’s lap at a distance of approximately 55 cm from a 17.5 inch screen. A 5-point infant calibration sequence was used to calibrate a Tobii 1750 remote eye tracker. During this procedure, a looming circle with accompanying sound was displayed in the 4 corners as well as the centre of the screen until the infant changed their looking direction. If this stimulus failed to capture the infant’s attention, then a brief animated video clip was shown at the calibration location instead of the looming circle. Calibration was repeated up to three times or terminated as soon as 5 points were calibrated successfully. All infants included in the analysis had a minimum of 4 good calibration points.

After the calibration sequence, and in *all* conditions, a soft piano tune started playing in the background at low volume, and remained on for the duration of the experiment. This was introduced as standard procedure in the lab following the discovery that the presence of low-level background music reduced the number of infants that became fussy in a study, as compared to completely silent studies. Presumably the dimmed lighting and complete silence (in a sound attenuated room) is off-putting to the infants because it is extremely unnatural.

Infants were presented with a sequence of eight familiarization trials, showing one object for 5000 ms each. In the Label and No Label condition, auditory stimuli (Label condition: “Look at the Timbo!” or “Do you see the Timbo?”; No Label condition: “Look at this!” or “Do you see this?”) began 500 ms after the onset of the visual exposure. This meant that in the Label condition the label itself (i.e., “Timbo”) was only heard 1020 ms after the start of the trial. Prior to Trial 1 and between all following trials, a 1-second attention-getter was shown (i.e., a small animated object was displayed at the centre of the screen while a chiming sound was played simultaneously). This was to ensure infants’ attention was directed at the display at the start of each new trial.

The background music, speech stimuli and sounds accompanying attention-getters were all played from the same pair of stereo speakers placed below and immediately to the left and right of the screen.

After the familiarization phase, infants were presented with five test trials, only the first of which is relevant for the present analysis and is termed the category boundary test. This consisted of a test stimulus pair as described above, i.e. a novel Timbo-object (possessing ball, claw and shell) side by side with a modified Timbo (possessing ball, claw and a novel part or ball, shell and a novel part), and was displayed for 10,000 ms. No auditory stimuli were provided during the test phase in any of the conditions.

Throughout the familiarization and test phases, infants’ eye movements were recorded by the Tobii 1750 eye tracker sampling at 60 Hz.

#### Data scoring

Infants’ eye gaze was processed by the Tobii Clearview fixation filter. Since unambiguous boundaries for areas-of-interest are hard to define a priori *across various stimuli*, each fixation location was assigned manually to one of the three object parts (claw, shell or ball) or the “background”. The construction of the stimuli with spatially separated object parts allowed a precise evaluation of which object parts infants were fixating at any instance. To this end, a Matlab script plotted the location of each fixation as calculated by the Tobii filter onto the target stimulus. A fixation (with start and duration times as in the Clearview output) was scored as falling on an object part if its location was on the object part itself or within 0.5 cm of the object part’s outline. Fixations falling outside of the three object parts (i.e. assigned to the “background”) were disregarded in the looking pattern analyses. A second independent observer scored thirty percent of all trials. Correspondence between the two sets of scores was very high (Pearson correlation r = .94).

## Results

Here, we first discuss global measures of looking time and looking proportions across the entire presentation time for category formation and boundary test trials respectively, both established measures of category learning in infants. Secondly, we describe infants’ responses with regard to individual object parts as learning unfolds during category formation, as a separate analysis.

### 

#### Total looking time during the familiarization phase

Average looking times accumulated during the first half of the familiarization phase vs. the second half in the different conditions are given in Table 1. A mixed ANOVA with Block (Trials 1–4 vs. 5–8) as a within-subjects factor, and Auditory Condition (Visual-only, Label, No Label) and Age (8-month-olds vs. 12-month-olds) as between-subjects factors revealed a main effect of Auditory Condition (*F*(2, 99) = 3.21, *p*<.05). No other significant effects were found. Specifically, there was no main effect of Block (*F*(1, 99) = 0.635, *p* = .427). The lack of significant decrease in looking during familiarization is not surprising given the “attention getters” placed between individual trial presentations. Even without the attention getter, it is not uncommon for young infants to sustain looking across familiarization trials when presented with rich photographic stimuli [6].

**Table 1:** Mean looking times (in milliseconds) during the familiarization phase (Experiments 1 and 2).

	8-month-olds		12-month-olds	
Condition	Trials 1–4: *M (SE)*	Trials 5–8: *M (SE)*	Trials 1–4: *M (SE)*	Trials 5–8: *M (SE)*
**Visual-only**	2346 (219)	2127 (200)	2500 (219)	2346 (228)
**Label**	2829 (277)	2817 (280)	2631 (244)	2639 (263)
**No Label**	2575 (198)	2578 (250)	2937 (215)	2956 (203)
**Sound (Exp. 2)**	-	-	2569 (250)	2325 (181)

Planned comparisons were carried out to investigate looking time differences across the conditions. These revealed that infants in the No Label condition (average across trials 1–8: *M* = 2756 ms, *SE* = 144 ms) looked at the objects significantly more than those in the Visual-only condition (*M* = 2325 ms, *SE* = 136 ms; *t*(67) = 2.18, *p*<.05, independent samples t-test, two-tailed). The difference between the Visual-only and Label condition (*M* = 2732 ms, *SE* = 175 ms) also approached significance (*t*(69) = 1.84, *p* = .070), indicating that infants in the conditions with verbal input in general had longer looking times than those in the Visual-only condition.

#### Preferential looking during category boundary test trial

On average, 12-month-olds spent 4443 ms (*SE* = 331 ms) gazing at the test display, while 8-month-olds spent 4119 ms (*SE = *353 ms). The minimum looking time on this trial was 458 ms. An ANOVA with factors Age and Condition revealed no significant effects (all *F*s<2.0, *p*s>.14).

A novelty preference score for the category boundary test (see Table 2) was calculated for each subject by dividing the time the infant spent fixating the novel object (ball, claw/shell, and novel part) by the time the infant spent fixating either of the two objects. Four 12-month-olds and 9 8-month-olds looked at only one of the target objects. These were included in the analysis since it was highly unlikely that they had not seen that there was a second object, considering the small display size. Planned comparisons (one-sample t-tests against chance = 0.5) were conducted for each condition. This revealed that only the 12-month-olds in the two conditions with speech input systematically preferred the novel out-of-category objects. Twelve-month-olds in the Label and in the No Label conditions performed at highly similar levels (Label: *M* = .62, *SE* = .05, *t*(15) = 2.21, *p*<.05; No Label: *M* = .64, *SE* = .05, *t*(15) = 2.52, *p*<.05; all two-tailed). Eight-month-olds exhibited no reliable novelty preference in any of the conditions, nor did the 12-month-olds in the Visual-only condition, suggesting that these groups failed to form a detailed category of Timbos.

**Table 2:** Mean looking proportions for the out-of-category test object during the category boundary test of Experiments 1 and 2, and statistical results.

	8-month-olds		12-month-olds	
Condition	*M (SE)*	*t*	*M (SE)*	*t*
**Visual-only**	.48 (.07)	*t*(14) = .325	.46 (.06)	*t*(14) = .6
**Label**	.55 (.07)	*t*(15) = .69	.62* (.05)	*t*(15) = 2.21
**No Label**	.44 (.08)	*t*(16) = .89	.64* (.05)	*t*(15) = 2.52
**Sound (Exp. 2)**	-	-	.49 (.06)	*t*(19) = 0.16

*Note.* Test results are based on one-sample t-tests against chance (two-tailed). Proportions marked with an asterisk were significantly different from chance at the.05-level.

In order to determine whether success on the category boundary test can be explained by the amount of attention directed at the stimuli alone, we performed correlations between the total familiarization looking time and the preference scores on test. For the 12-month-olds these data were not correlated (*r* = .017, *p*>.9), indicating that it is not due to changes in attention alone that these infants either did or did not exhibit novelty preference on test. For the 8-month-olds, there was a weak correlation (*r* = .29, *p* = .047). Taken together with the lack of novelty preference found across the 8-month-old groups, this suggests that (partial) category encoding at 8 months depends more on how long infants spend engaging with the visual stimuli, than on labeling.

#### Gaze patterns during the familiarization phase

In order to investigate whether adding labels increased infants’ attention to the low-variability part during learning we obtained looking proportions by dividing the accumulated looking time for the ball by the accumulated looking time for the three object parts (ball, claw and shell) for each trial and each participant. We further split the familiarization phase into two blocks of 4 trials as above. The looking proportions directed at the low-variability part for blocks 1 and 2 were then subjected to a mixed design ANOVA with within-subjects factor Block and between-subjects factor Condition. This revealed no significant effects (all *F*s<.42, *p*s>.66). There was, in particular, no effect of Condition (*F*(1,47) = .265, *p* = .769), nor an interaction of Block×Condition (*F*(2,47) = .417, *p* = .661).

While this result reflects the conventional way of looking at familiarization, examining gaze patterns accumulated across whole trials is in fact rather coarse. After all, studies investigating the time course of category assignment find effects within a few hundred milliseconds after the onset of stimulus exposure [37], [38]. It seems likely that obtaining looking proportions over a window of 5000 milliseconds may obscure effects present in the data, which are fast responses to incoming information. We therefore divided the trials into 1000 ms windows in order to compare gaze patterns in the three conditions in more detail.

**Figure 2:**
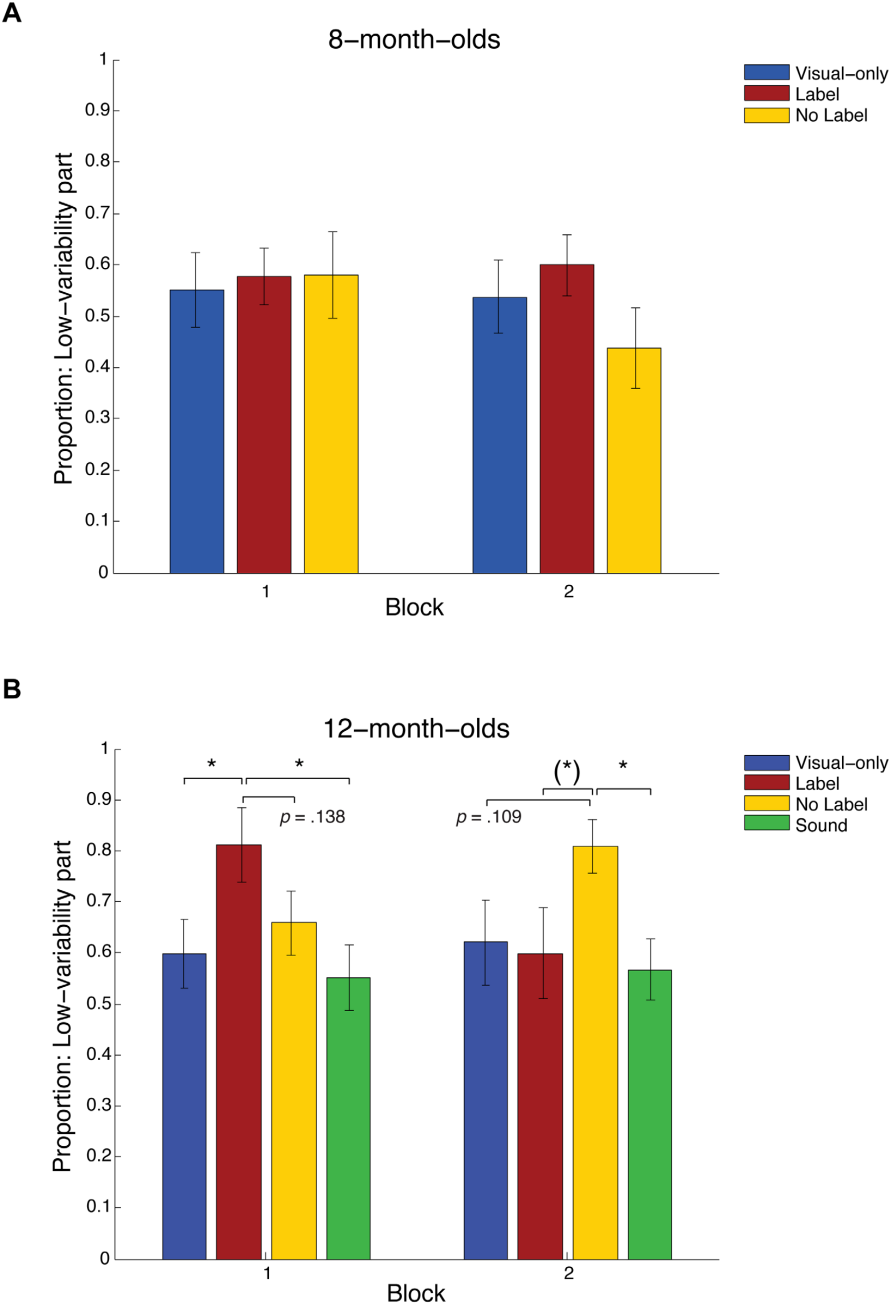
Time course of a trial and analysis window. The main analysis focuses on initial responses (1–1000 ms) to the visual stimulus, which reflect categorization processes.

We were particularly interested in the first of the time windows. As described above, recognizing an object as part of a category is a fast, immediate process that occurs within a few hundred milliseconds of stimulus onset. If labels have an impact on the process of category learning, a stimulus should be treated differently *from the start of the trial* if similar stimuli have been labeled in the past (in other words, recognizing a new object from the target category should be different if other category members have previously been encountered together with the label “Timbo”, in comparison to not having heard labels). Focusing on the first portion of each trial should therefore allow us to isolate categorization-related behavior. The division into five 1000 ms windows meant that the first of these windows corresponded to the proportion of the trial that occurred before the novel label (in the Label condition), i.e. a point in time when infants were – in terms of information – relying solely on the visual image (see Figure 2 for an illustration of the trial time course).

**Figure 3:**
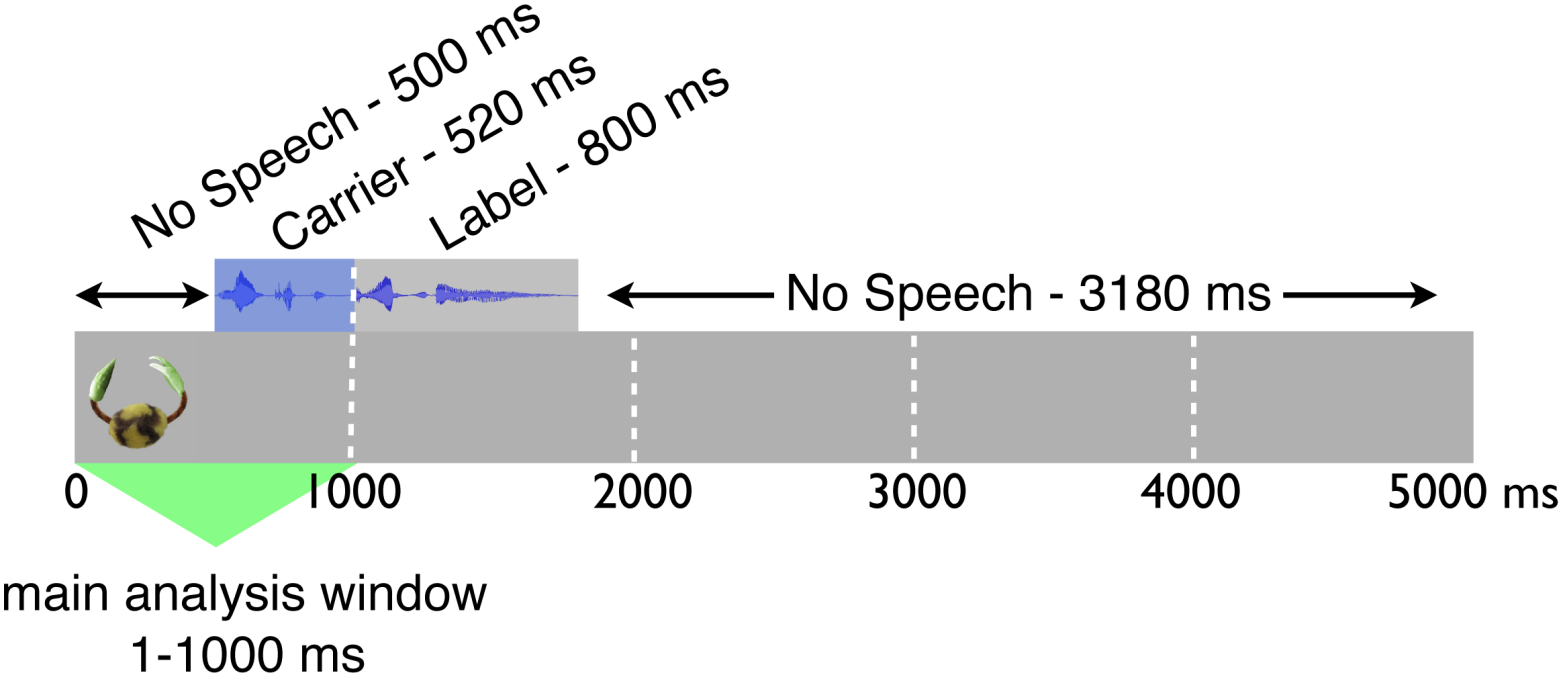
Looking at the low-variability part during familiarization. Looking proportions measured for the low-variability object part (ball) during the first 1000 ms of each familiarization trial, averaged across Block 1 (Trials 1–4) and Block 2 (Trials 5–8): A. data from 8-month-olds (Experiment 1), B. data from 12-month-olds (Experiments 1: Visual-only, Label and No-Label conditions & Experiment 2: Sound condition). Black bars represent standard errors.

As an initial analysis of all five 1000 ms windows (again averaged over two blocks of four trials) we conducted separate mixed design ANOVAs with factors Block, Age and Condition for each window. For the first time window, 1–1000 ms, this revealed a highly significant interaction of Block×Age×Condition (F(2,98) = 5.89, p = .004). Average looking proportions for this time window are shown in Figures 3A and 3B for Blocks 1 and 2 and both age groups.

By contrast, there were no three-way interactions in windows 2–5 (1001–5000 ms; *F*s<.79, *p*s>.17). This confirmed our hypothesis that categorization-relevant processes occur at the start of visual exposure, whereas looking later on in the trial represents exploration rather than recognition.

Since we will in the following focus on the first time window, 1–1000 ms after trial onset, and the first label did not occur until after this window in Trial 1, it is important to establish that infants’ looking across the conditions did not already differ at the very beginning of familiarization. An ANOVA on the trial 1 (window 1) data with factors Age and Condition confirmed that this was not the case (all *Fs*<.52, *ps*>.47).

To explore the 3-way interaction between Block×Condition×Age further, both age groups were subjected to separate mixed design ANOVAs with factors Block (1, 2) and Auditory Condition (Visual-only, Label, No Label). For the 8-month-olds this revealed no significant effects (all *F*s<1.2, *p*s>.31). For the 12-month-olds, however, the ANOVA revealed a highly significant interaction of Block×Auditory Condition (*F*(2,47) = 6.224, *p* = .004). No other effects were significant (all *F*s<.97, *p*s>.38). Planned contrasts showed that in Block 1 (Trials 1–4), the 12-month-olds in the Label condition looked more at the low-variability part (ball) than those in the Visual-only condition (*F*(1,47) = 4.634, *p* = .037). This confirms the hypothesis put forward by Waxman and Markow: infants’ fixations in the Label condition were focused on the “common” parts that were most similar between exemplars. The difference in looking at the low-variability parts in the Label vs. the No Label condition approached significance for Block 1 (*F*(1,47) = 2.274, *p* = .138). The No Label and Visual-only conditions did not differ (*F*(1,47) = .374, *p* = .544). In Block 2, by contrast, infants in the No Label condition exhibited more looking at the low-variability part than those in either the Label (marginally significant: *F*(1,47) = 3.298, *p* = .076) or the Visual-only condition (approaching significance: *F*(1,47) = 2.667, *p* = .109).

## Discussion

Our study found that 8-month-olds did not learn the Timbo category in silence, and this did not change in the presence of labeling or non-labeling phrases. In fact, only 12-month-olds who had received speech input (with or without labels) successfully formed a category over the Timbo stimuli that allowed them to recognize a novel part substituted for either the claw or shell. While this confirmed the hypothesis that labels facilitate categorization, the finding that a similar effect can be achieved by phrases not containing any novel labels was unexpected. This contrasts with previous research (e.g., [15]) in which it was reported that facilitation of categorization was specific to novel labels. In particular, this finding raises the question to what extent facilitation is caused by an increase in attention due to the presence of an auditory signal, as opposed to labeling specifically. Although the part-based results from the familiarization phase suggest differences in processing between the two conditions with speech input, whether *any* type of sound facilitates category learning is an important question that is yet to be addressed. For this reason, we introduced an additional control condition in which 12-month-olds were presented with non-linguistic sounds instead of spoken phrases alongside the visual stimuli. Based on previous findings [16] we did not expect successful categorization in this condition.

## Experiment 2

### Method

#### Participants

Twenty 12-month-olds (mean age: 371 days; 15 girls) participated in this study. Two additional infants completed the study but were not included in the analysis due to calibration error (1 infant) or failure to reach the looking criterion (1 infant). All infants heard English as their only language at home.

#### Stimuli, Design and Procedure

The visual stimuli were the same as in Experiment 1. The auditory stimulus was an artificial sound (sounding like a “laser” from a computer game) that has previously been used as an unfamiliar, non-linguistic auditory stimulus for infants at this age [25]. The length of the sound was matched to the length of the label (800 ms).

The procedure was identical to that in Experiment 1, with the exception that on all eight familiarization trials infants heard the non-linguistic sound described above, which began 1000 ms after trial onset.

## Results

As for Experiment 1 above, we first provide global measures of looking time during the familiarization phase and test trial, and then report the more detailed analysis of looking patterns with regard to object parts across category formation.

### 

#### Total looking time during familiarization

Mean looking times exhibited during the first and second half of the familiarization phase are listed in Table 2. A two-tailed, paired t-test revealed that there was no decrease in looking between the two blocks (*t*(19) = 1.02, *p*>.32). A one-way ANOVA with factor Condition showed that looking time did not differ from the 12-month-olds in Experiment 1 (*F*(3,66) = 1.39, *p*>.25).

#### Preferential looking during category boundary test trial

Novelty preference scores were calculated as in Experiment 1. A two-tailed t-test against chance revealed that infants’ looking at the novel object in the Sound condition did not exceed chance (*t*(19) = 0.16, *p*>.87).

#### Gaze patterns during the familiarization phase

Looking proportions for the object part “ball” during the first 1000 ms window of each familiarization trial were calculated as described for Experiment 1, and averages obtained for Blocks 1 (Trials 1–4) and Block 2 (Trials 5–8). The results are shown in Figure 3B. Planned comparisons with the conditions from Experiment 1 showed that infants’ looking patterns across the whole familiarization phase were similar to those in the Visual-only condition. The only significant differences were between the Sound and Label condition in Block 1 (*F*(1,66) = 7.364, *p* = .008), and between the Sound and No Label condition in Block 2 (*F*(1,66) = 5.248, *p* = .025; all remaining contrasts: *F*s<1.21, *p*s>.28).

## General Discussion

Experiment 2 confirmed our hypothesis that the facilitation of category formation in 12-month-olds is not achieved by non-linguistic sounds. This implies that the findings from Experiment 1– facilitated category formation in the presence of labeling as well as non-labeling phrases – are not simply due to domain-general, attentional processes. Clearly these results are specific to speech.

Our results from 12-month-olds are as follows. First, labeling and non-labeling phrases facilitate categorization. In contrast, learning in the Visual-only and Sound conditions is unsuccessful. Secondly, eye movement patterns exhibited by infants in the Label and No Label conditions during familiarization are different, meaning that although both stimuli lead to success they achieve this in different ways. In particular, infants in the Label condition exhibited a commonality preference already at the beginning of familiarization, whereas infants in the No Label condition exhibited a similar preference for the least variable part during the second part of familiarization. We can therefore conclude that labels have a unique effect that is not achieved with other linguistic or non-linguistic stimuli.

Our result is consistent with Waxman and Markow’s [15] hypothesis that labels “highlight commonalities”: infants appeared to have a preference for the part that was most consistent across exemplars. This early commonality focus is clearly neither a response to speech in general, nor to novelty in general, but specific to speech containing a novel label. The preference for the low-variability part disappeared, however, within the course of a few object presentations. The reason for this could simply be novelty preference: while the orienting to the most similar part is a strong initial effect, infants may soon become familiarized with this object part in particular – it is, after all, the part that changes least. The short duration of commonality preference is therefore not surprising.

Are our results consistent with any of the mechanisms we identified above (i.e.; a. auditory overshadowing, b. increased saliency and thereby increased attention, or c. high-level cognitive effects that involve re-activating previously formed representations)?

At a first glance, spending more time on a low-variability part appears like more conservative behavior, or even familiarity preference. A familiarity preference during learning would indicate that cognitive load on labeled trials is so high that infants continue to process the most predictable part rather than being able to move on to the more novel elements – a hypothesis that is in line with auditory overshadowing (Hypothesis a.) [25]. However, at a macro-level our experiment found no evidence for auditory overshadowing. On the contrary, infants in the Label condition (as well as the No Label condition) outperformed infants in the Visual-only condition as far as the category boundary test is concerned. Infants in the Sound condition (Experiment 2) did not exhibit an obvious deterioration in performance either, although the lack of familiarity of the sound used in this condition should have produced the most disruptive overshadowing effect [26]. Given the subtle difference between the out-of-category object in the category boundary test and the target category, it seems unlikely that familiarization in the Label and No Label conditions could have been successful precisely *because* auditory input overshadowed the detailed encoding of visual stimuli. While overshadowing may reduce dissimilarities between exemplars [27], the presence of such a mechanism would surely have made it harder for the infants to distinguish between the out-of-category object and the Timbos. In that case, no preferential looking should have occurred.

That said, it should be kept in mind that our studies were *not* designed to examine specifically whether auditory overshadowing occurs or not. The possibility that there may have been some amount of auditory overshadowing in *all* conditions in our study due to the presence of the low-volume, soothing background music cannot be completely excluded. Nevertheless, our results show clearly that if such overshadowing took place, then it was overridden by the effect of labels (and indeed that of non-labeling phrases).

Is it possible that overshadowing processes occurred just at the beginning of learning? Perhaps such initial limitation of learning could produce a commonality focus that structures the learning process to first incorporate low-variability parts, and *then* move on to the more difficult, high-variability parts. However, in this case it would be hard to explain the behavior occurring in the No Label condition. As overshadowing effects should be less strong with only familiar linguistic stimuli this would leave the *late* commonality focus in this group unexplained. For this reason we can conclude that auditory overshadowing was not the core factor underlying the facilitation effects we observed.

If an increase in saliency through the presence of spoken language is the reason for the facilitative effects we observe in the 12-month-olds (Hypothesis b.), then this must be a domain-specific effect rather than based on general increased attention in a multimodal scenario. Otherwise infants should also have learned the target category in the Sound condition (Experiment 2). It is possible that infants are, by the age of 12-months, tuned into treating speech (more than other auditory stimuli) as a communicative, intentional signal – even new-born infants exhibit a preference for speech over non-speech [39]. Let us therefore assume that both stimuli (labeling and non-labeling phrases) increase the saliency of the target objects, and infants therefore successfully encode a category (in other words, both effects *share* a mechanism). Why, then, do they exhibit different looking patterns during familiarization? It seems plausible that non-labeling phrases increase attention by just enough to allow infants to extract commonalities after 5–8 exposures, but novel labels increase attention even more, allowing for a very rapid commonality focus to occur.

An alternative explanation for the different looking patterns during familiarization is that the mechanisms underlying learning in both conditions is in fact qualitatively different and learning with labels involves the re-activation of representations of previously encountered stimuli (Hypothesis c.). In particular, it is possible that the infants in the Label condition engaged in what may be an early precursor of implicit naming [34]. Having just heard a name for a novel, exciting object, the similarity match between the ball part in the previous (named) object(s) and the ball part in the new object that has just appeared on the screen may elicit a categorization response that involves triggering linguistic processes. Even though *word learning* as such probably does not occur in these early trials of the familiarization phase, precursors of retrieving speech code relating to the perceived object may be involved.

To summarise, the looking patterns we observe are consistent with the Hypothesis (b.) of increased saliency, involving identical underlying mechanisms for learning with labeling and non-labeling phrases, albeit with quantitative differences that result in the different looking patterns during familiarization. Our results are also consistent with the hypothesis of re-activation of previous visual representations in the presence of labels (c.), meaning that qualitatively different mechanisms would be responsible for learning in the two conditions. While the two hypotheses are not necessarily mutually exclusive (bottom-up saliency and top-down mechanisms could work simultaneously), our study does not allow us to discriminate them – this is subject to further work.

Regardless of what the underlying mechanism for learning is, the facilitation effects we find for the two conditions involving language must be due to an improved category representation that is established in these cases, but not when learning in silence. Why does a commonality focus lead to such an improvement? We believe that a commonality focus can be seen as an indicator that comparison occurs across different category instances. Recognizing commonalities may, for instance, allow for tighter grouping of category exemplars [40]. Clearly, however, 12-month-olds in the Label and No Label conditions did not just learn about the commonalities, but also about the high-variability features: it is impossible to exhibit novelty preference on test without representing the claw and shell parts. One possibility is that having extracted the commonality – and thereby establishing category membership more easily – allows infants to more reliably assume that different-looking variable parts are instances of the same feature, and thereby form a more accurate representation of these object parts.

One discrepancy between our study and previous work needs to be addressed. Why did infants in the No Label condition outperform those in the Visual-only condition in the category boundary test? From an information-theoretic point of view it is hard to conceive of the non-labeling phrases as “informative”. Thus, infants in this condition were not expected to benefit from the speech input, and indeed, previous research has found that infants in a No Label condition performed less well than infants in a Label condition (e.g., [15]). As discussed above, one hypothesis is that hearing a communicative phrase (highlighting the relevance of the current visual display) may have enhanced infants’ attention, and thereby their visual processing in comparison to the Visual-only group. This may have led to the increased categorization performance we observed in the test trial. During the first block of the familiarization phase, infants in the No Label condition behaved similarly to those in the Visual-only condition. It was not until later that they began to exhibit a preference for the low-variability part, which was significantly greater than for the other conditions in Block 2. It seems plausible that this reflects a gradual “extraction” of the ball as the most consistent part across different stimuli. One crucial difference between our study and previous work by Waxman and colleagues (who did not find facilitation of categorization in the presence of non-labeling phrases) is the novelty of our stimuli. Attention-directing phrases like “Look at this!” may not be useful when infants are confronted with known objects (such as rabbits, or even dinosaurs, which at least fall into a familiar global category of “animals” that they share similarities with) for which they are likely to activate a previously formed category representation. For novel objects, by contrast, there is no previous representation to be accessed, so attention-directing phrases perhaps achieve a focus that is just enough to extract similarities between successive items over time.

A surprise was that the 8-month-olds did not exhibit a novelty preference in the test trial even in the presence of labels. Previous research indicated that labels can facilitate categorization at the much younger age of 3 to 4 months [19]. This may perhaps be attributed either to the novelty of our stimuli or their perceptual richness. Previous studies investigating the interaction of labeling and categorization have often used familiar animal categories, or classes like dinosaurs [16], [19], which are not necessarily familiar, but bear more similarity to familiar animal categories than Timbos do. The Timbo images display clearly visible texture and depth, which contrasts with the line drawings used in other studies and may have contributed to the complexity of categorization.

In conclusion, we have shown that both labeling and non-labeling phrases can facilitate categorization in 12-month-olds. By contrast, 8-month-olds did not appear to benefit from either type of linguistic input to the same extent. We have furthermore provided evidence that labels rapidly direct 12-month-old infants’ attention to commonalities between exemplars. To our knowledge, this is the first time this has been observed directly by measuring eye movements. Our analyses of fixations during the familiarization phase of the experiment showed that phrases containing novel labels and phrases not containing novel labels had different effects on infants’ visual processing. Even though both types of auditory input facilitated categorization, only the infants hearing novel labels exhibited a focus on the common object part during the first block of familiarization. We therefore conclude that the learning processes in both cases are different and only novel labels immediately direct attention to commonalities.
